# Detection and Molecular Characterization of *Giardia* and *Cryptosporidium* spp. Circulating in Wild Small Mammals from Portugal

**DOI:** 10.3390/ani13030515

**Published:** 2023-02-01

**Authors:** Laura Lux, Rainer G. Ulrich, Sérgio Santos-Silva, João Queirós, Christian Imholt, Christian Klotz, Joana Paupério, Ricardo Pita, Hélia Vale-Gonçalves, Paulo Célio Alves, João R. Mesquita

**Affiliations:** 1 University of Greifswald, Domstraße 11, 17489 Greifswald, Germany; 2 Institute of Novel and Emerging Infectious Diseases, Friedrich-Loeffler-Institut (FLI), Federal Research Institute for Animal Health, Südufer 10, 17493 Greifswald-Insel Riems, Germany; 3 Instituto de Ciências Biomédicas Abel Salazar (ICBAS), Universidade do Porto, Rua de Jorge Viterbo Ferreira, 228, 4050-313 Porto, Portugal; 4 CIBIO—Centro de Investigação em Biodiversidade e Recursos Genéticos, InBIO Laboratório Associado, Campus de Vairão, Universidade do Porto, 4485-661 Vairão, Portugal; 5 BIOPOLIS Program in Genomics, Biodiversity and Land Planning, CIBIO, Campus de Vairão, 4485-661 Vairão, Portugal; 6 Institute for Epidemiology and Pathogen Diagnostics, Julius Kühn-Institute (JKI), Federal Research Centre for Cultivated Plants, 48161 Münster, Germany; 7 Unit 16 Mycotic and Parasitic Agents and Mycobacteria, Department of Infectious Diseases, Robert Koch-Institute, 13353 Berlin, Germany; 8 MED—Mediterranean Institute for Agriculture, Environment and Development, CHANGE—Global Change and Sustainability Institute, IIFA—Institute for Research and Advanced Training, UBC—Conservation Biology Lab, University of Évora, Mitra Pole, Ap. 94, 7006-554 Évora, Portugal; 9 Laboratório de Ecologia Fluvial e Terrestre, CITAB—Centro de Investigação e Tecnologias Agroambientais e Biológicas, Universidade de Trás-os-Montes e Alto Douro, 5001-801 Vila Real, Portugal; 10 Estação Biológica de Mértola, 7750 Mértola, Portugal; 11 Epidemiology Research Unit (EPIUnit), Instituto de Saúde Pública da Universidade do Porto, Rua das Taipas 135, 4050-600 Porto, Portugal; 12 Laboratório para a Investigação Integrativa e Translacional em Saúde Populacional (ITR), Rua das Taipas 135, 4050-600 Porto, Portugal

**Keywords:** *Cryptosporidium muris*, *Giardia microti*, sequence typing, rodent, shrew, reservoir

## Abstract

**Simple Summary:**

Wild small mammals can be a veterinary and public health concern, because they can act as reservoir hosts for numerous pathogens and potentially transmit them to humans, domestic animals and other wildlife species. This study represents the first investigation of the diarrhea-causing parasites *Cryptosporidium* spp. and *Giardia* spp. in wild rodents and shrews from Portugal. *Cryptosporidium* spp. was rarely and *Giardia* was frequently detected in the feces of the analyzed species, with the southwestern water voles (*Arvicola sapidus*) and Lusitanian pine voles (*Microtus lusitanicus*) showing the highest infection rates of *Giardia* spp. Genetic characterization based on common genomic marker sequences revealed the rodent-adapted *Giardia microti* and potentially zoonotic *Cryptosporidium muris* as the only circulating species. These findings suggest the limited role of wild rodents and shrews as natural sources of human infections in Portugal regarding the investigated parasites. Moreover, the host ranges of *Giardia* and *Cryptosporidium* spp. were extended and the obtained genetic sequences of *Giardia microti* are useful for future comparative studies. From the One-Heath perspective, this study helps to understand the epidemiology of *Giardia* spp. and *Cryptosporidium* spp. in wildlife.

**Abstract:**

*Cryptosporidium* spp. and *Giardia* spp. are important diarrhea-causing protozoan parasites worldwide that exhibit broad host ranges. Wild small mammals can harbor host-adapted and potentially zoonotic species of both parasites. The aim of this study was to investigate *Cryptosporidium* spp. and *Giardia* spp. in wild rodents and shrews in Portugal, focusing on the protist’s occurrence and genetic diversity. Molecular screening by PCR at the small subunit (*SSU*) rRNA gene locus of 290 fecal samples from wood mice (*Apodemus sylvaticus*), southwestern water voles (*Arvicola sapidus),* Cabrera’s voles *(Microtus cabrerae),* Lusitanian pine voles (*Microtus lusitanicus),* Algerian mice (*Mus spretus)* and greater white-toothed shrews (*Crocidura russula*) in Northeast Portugal revealed the low occurrence of *Cryptosporidium* spp. (1%) and high occurrence of *Giardia* spp. (32.8%). The analysis revealed that “species” was the only significant factor associated with the increasing probability of *Giardia* spp. infection, with the highest prevalence reported in southwestern water voles and Lusitanian pine voles. *Cryptosporidium* and *Giardia* species determination at the *SSU* rRNA gene locus revealed *C. muris* and *G. microti* as the only circulating species, respectively. Subtyping of the glutamate dehydrogenase (*gdh*) and beta-giardin (*bg*) genes provided evidence of the high genetic diversity within the *G. microti* clade. This study suggests that rodent-adapted *G. microti* occurs to a large extent in cricetid hosts and supports the limited role of wild rodents and shrews as natural sources of human infections in Northeast Portugal regarding the investigated parasites. Moreover, this is the first record of *G. microti* in southwestern water voles, Lusitanian pine voles, Algerian mice, wood mice and Cabrera’s voles and *C. muris* in Cabrera’s voles. Finally, this study improves the database of sequences relevant for the sequence typing of *G. microti* strains and provides new insights about the epidemiology of *Giardia* spp. and *Cryptosporidium* spp. in wild rodents and shrews, two parasite genera of high importance for public and animal health.

## 1. Introduction

Rodents (order Rodentia) are the most abundant and widely distributed mammalian group, adapted both to wild and anthropogenically modified habitats [1]. Other small mammals, such as shrews and other insectivores (order Eulipotyphla) are also widespread in the world [2]. These small mammals are considered to have an important impact on public health, acting as reservoirs of zoonotic bacterial, viral and parasitic agents in the environment and contributing to food, water and soil contamination [3]. Among the pathogens transmitted by small mammals, parasites tend to receive less attention [4,5]. These include the diarrhea-causing protists of the genera *Giardia* and *Cryptosporidium* that are among the most common enteric parasites of humans and domestic animals, transmitted mainly by the fecal–oral route, and are increasingly recognized in a diverse range of wildlife species, including rodents and insectivores [6,7,8].

The flagellate protozoon *Giardia duodenalis* (synonyms *Giardia intestinalis* and *Giardia lamblia*) can cause giardiasis, a disease characterized by gastrointestinal and diarrheal symptoms, affecting humans, domestic animals, and wildlife across the world [9,10]. *G. duodenalis* is currently classified as a species complex with eight assemblages (A to H) based on the sequence analyses of genetic markers, such as glutamate dehydrogenase (*gdh*), triosephosphate isomerase (*tpi*), beta-giardin (*bg*) and the small subunit (*SSU*) rRNA gene [11,12]. The assemblages display different host preferences. Among them, assemblages A and B show the broadest host range and are considered to be potentially zoonotic [9,13], occurring in humans and many other mammals, including rodents and insectivores [7,11]. Assemblages C and D are mainly found in canids, assemblage E in hoofed animals, assemblage F in cats, assemblage G in rodents (in particular rats), and assemblage H in pinnipeds [12,14]. In addition to *G. duodenalis,* at present, two other *Giardia* species, *Giardia microti* and *Giardia muris,* have been detected in rodents with supposedly distinct host specificities. It is speculated that *G. microti* and *G. muris* chiefly infect rodents that belong to the families Cricetidae and Muridae, respectively [13,15,16,17]. Hence, rodents are potentially parasitized by different *Giardia* species, namely *G. microti*, *G. muris* and assemblages A, B and G of *G. duodenalis*. Moreover, the potential zoonotic risk of *Giardia* spp. has been highlighted by several studies [3,4,18,19], which mainly refer to larger rodents, such as the North American beaver (*Castor canadensis*) [20,21,22] and pet chinchilla (*Chinchilla lanigera*) [18,23,24]. The role of smaller rodents, commonly found in natural and human habitats, in zoonotic transmission is not well-resolved [25]. Systematic genotyping surveys that examine the *Giardia* species in different wild rodents and insectivores are rare [7,17,19,26,27,28] and the distribution of the *Giardia* species in the wide range of small mammal genera and their potential zoonotic risk remain to be clarified [29].

The intracellular protozoa of the genus *Cryptosporidium* exhibit a wide genetic diversity and host range [2]. At least 44 *Cryptosporidium* species are considered to be taxonomically valid [30], distinguishable by genomic markers such as *SSU* rRNA and *gp60* genes [31]. *Cryptosporidium hominis* and zoonotic *C. parvum* are responsible for most of the globally reported cryptosporidiosis cases in humans, and are recognized as a leading cause for diarrhea and mortality in young children and immunocompromised individuals [32,33,34]. Other less common *Cryptosporidium* species, e.g., *C. meleagridis*, *C. felis*, *C. canis*, *C. viatorum*, *C. muris, C. ubiquitum* and *C. cuniculus,* have been reported in humans [35], as well as in various mammal species [36,37]. Besides farm animals, rodents and insectivores are considered as important reservoirs of *Cryptosporidium* spp. [38,39]. A large variety of *Cryptosporidium* spp. has been described in wild rodents, with 35 distinct *Cryptosporidium* spp./genotypes reported in Europe [40]. Most of the *Cryptosporidium* species and genotypes harbored by rodents and insectivores are host-specific or show a relatively narrow host range [41]. Specific associations include, e.g., *C. alticolis, C. microti,* and vole genotypes I–VII in voles, *C. apodemi* in mice, *C. ratti* and mouse genotype II in rats, *Apodemus* genotypes I and II in *Apodemus* spp. and shrew genotypes I and II in shrews [2,41]. In contrast, other *Cryptosporidium* species identified in rodents display broader host ranges that include humans and various other animals (e.g., *C. parvum, C. ubiquitum, C. muris and C. meleagridis*) [19,33,36]. The fact that the latter species with broad host specificities are commonly detected in humans emphasizes that small mammals may contribute to the zoonotic transmission of *Cryptosporidium* spp. by excreting infectious oocysts [41,42]. However, to date, there is no direct evidence for the transmission pathways of rodent-borne *Cryptosporidium* at the human–animal–environment interface [41].

Molecular studies on *Giardia* spp. and *Cryptosporidium* spp. in wildlife frequently report the presence of these parasites in rodents of different genera [17,41]. However, additional molecular-based epidemiological studies are needed to understand the circulation and host specificity of those pathogens and assess the risk of potential zoonotic events. While the occurrence of *Giardia* spp. and *Cryptosporidium* spp. in humans and drinking water samples has been extensively reported in Portugal [43,44,45,46,47], the role of rodents and insectivores as reservoirs of these protists has not been examined so far. Therefore, the objective of the present study was to investigate the occurrence and genetic diversity of *Cryptosporidium* spp. and *Giardia* spp. in fecal samples of wild rodents and shrews from Northeast Portugal.

## 2. Materials and Methods

### 2.1. Fecal Sample Collection

The fecal sample collection and molecular analyses for the taxonomic identification of small mammal species are described in detail in the work of Barão et al. [48]. Samples were initially collected to assess if highly patchy and heterogeneous mosaics of different land uses associated with olive groves in Northeast Portugal allow for the occurrence of a rich small mammal community, including high occupancies (including species of conservation concern), and low occupancy rates by potential pest species. Briefly, passive sampling took place in spring 2020 (May to July) in the Trás-os-Montes region, located in Northeast Portugal. The landscape of this region is largely dominated by olive groves and exhibits generally high biodiversity across different taxonomic levels, including small mammals [48,49]. The sampling units were represented by land mosaics that correspond to small farm units operating independently from each other. Each sampling unit was centred on a focal olive grove patch and surrounded by different habitats (shrubs, other fruit trees, pastures, forest, and agriculture areas) [48]. As the sampling units were >1200 m apart from each other, sampling units were classified to be independent, given the typical dispersal distances of small mammal species, e.g., 500 m for wood mice [48,50]. Small mammal non-invasive sample collection in each sampling unit consisted of 40 m long transects for detecting presence signs of small mammals and collecting fecal samples. The number of transects in each sampling unit was defined according to its size (4 transects/ha), ranging between 7 and 19 transects per sampling unit. Transects were geo-referenced in the field using a GPS device and fecal samples were collected into 1.5 mL microtubes and covered with 98% alcohol. For each sample collected, sterilized sampling material and new latex gloves were used. For this study, 290 fecal samples of six wild small mammal species from twenty-four different sampling units and six different habitat types were included. The samples originated from one insectivore (greater white-toothed shrew, *n* = 47) and five species of small rodents, including two species of the family Muridae (wood mouse, *n* = 43; Algerian mouse, *n* = 48) and three species of the family Cricetidae (southwestern water vole, *n* = 52; Cabrera’s vole, *n* = 49; Lusitanian pine vole, *n* = 51), as determined by the genetic identification of the host species based on the fecal molecular analysis of a small fragment of the 12S rRNA gene [48].

### 2.2. Nucleic Acid Extraction

Lentil-sized pieces of feces were transferred into a microtube, containing phosphate-buffered saline (PBS; pH 7.2; final concentration 10% *w/v*) and 0.2 g sterile silicone microbeads (Precellys Lysing kits, Bertin Technologies SAS, Montigny-le-Bretonneux, France). Feces samples were shredded with plastic pestles and vortexed for 5 min using the cell disruptor Disruption Genie (Scientific industries, Inc., Bohemia, NY, USA). After 5 min of centrifugation at 8000×g, DNA extraction was carried out from 140 µL of the clarified supernatant using the automatic extraction machine QIAcube^®^ (Qiagen, Hilden, Germany) and QIAamp^®^ DNA Mini Kit (Qiagen, Hilden, Germany). DNA extracts were eluted in GRS PCR Grade Water (GRiSP^®^, Porto, Portugal) and preserved at −20 °C until further use.

### 2.3. General Procedure for PCR Amplifications

Samples were screened in pools of five samples (5 µL per sample) and unfolded in cases of producing amplicons of the expected size. For *Giardia* spp. detection, real-time (quantitative) PCR (qPCR) reactions were run on a CFX Connect Real-Time PCR Detection System (Bio-Rad, Hercules, CA, USA) with the Xpert Fast Probe (uni) (GRiSP^®^, Porto, Portugal). Afterwards, data were analyzed using the CFX Maestro 1.0 Software version 4.0.2325.0418 (Bio-Rad, Hercules, CA, USA). For *Giardia* spp. characterization and *Cryptosporidium* spp. detection and characterization, end-point PCR reactions were performed on a T100 thermocycler (Bio-Rad, Hercules, CA, USA) with 2x Xpert Fast Hotsart Mastemix (GRiSP^®^, Porto, Portugal). PCR thermocycling conditions were set accordingly to the original protocols previously published (see Table S1) and adjusted to the commercial PCR kits. For endpoint PCR after an initial cycle of 3 min at 95 °C (enzyme activation and denaturation of template DNA), 40 cycles were performed at 95 °C for 15 s, 15 s at the annealing temperature, and 72 °C for 2 s for extension purposes, with a final extension step of 72 °C for 3 min. For qPCR after an initial cycle at 95 °C for 3 min (enzyme activation and denaturation of template DNA), 40 cycles were performed at 95 °C for 5 s, and 30 s at the annealing/extension temperature. Modifications in the annealing temperatures are mentioned in the following sections. All PCR amplification products were visualized by electrophoresis on 1.5% agarose gels stained with Xpert Green DNA Stain (GRiSP^®^, Porto, Portugal).

### 2.4. Giardia spp. DNA Screening

To detect *Giardia* spp., a qPCR protocol was initially applied to amplify a 62-base pair (bp) region of the *SSU* rRNA gene [51]. A selection of the qPCR *Giardia* spp.-positive samples was reassessed by sequence-based multi-locus sequence typing (MLST) to determine the *Giardia* species. For the selection, only *Giardia* spp.-positive samples with qPCR cycle threshold (Ct) values ≤32 were chosen, considering at least 15% of the qPCR positive samples from each rodent species, different habitats, and lowest Ct values. For the MLST, the genes that encoded for the *SSU* rRNA, and GDH and BG proteins of the parasite were selected, because the applied *SSU* rRNA gene PCR assay showed amplification success in a study on *Giardia* spp. in rodents using modified primer sets [17] and the *gdh* and *bg* genes are among the most commonly used regions for the MLST of *Giardia* spp. [52]. To amplify the corresponding gene fragments by nested PCRs, the following external and internal primer pairs were used, respectively: for the *SSU* rRNA fragment, the primer pairs RH11 derivatives/Gia2150c and RH11 derivatives/RH 4 derivatives (expected product of 293 bp; [17]) were used, for the *gdh* gene fragment, the primer pairs GDHeF/GDHiR and GDHiF/GDHiR (432 bp, [53]) were used and for the *bg* gene fragment, the primer pairs G7/G759 and G99/G609 (511 bp, [54]) were used. The annealing temperatures were modified compared to the original protocols. For the *SSU* rRNA gene PCR, the temperatures were set at 56 °C (first round) and 62 °C (second round); for the *gdh* PCR, the temperatures were set at 57 °C (both rounds). As a positive control, one qPCR *Giardia* spp.-positive sample from this study was sequenced for validation (*G. microti*) and used from then on as a control in all qPCR and PCR protocols.

### 2.5. Cryptosporidium spp. DNA Screening

To detect *Cryptosporidium* spp., a nested PCR approach was used to amplify a partial region of the *SSU* rRNA gene (~587 bp, [55]). The external primer pair CR-P1/CR-P2 and the internal primer pair CR-P3/CPB-DIAGR were used. As a positive control, a *Cryptosporidium* spp.-positive sample (*C. scrofarum*) from wild boar (*Sus scrofa*) was used in all the PCR assays.

### 2.6. Sequencing and Sequence Analysis

PCR amplicons of the expected sizes of 293 bp (*Giardia* spp. *SSU* rRNA), 432 bp (*Giardia* spp. *gdh*), 511 bp (*Giardia* spp. *bg*) and 587 bp (*Cryptosporidium* spp. *SSU* rRNA), respectively, were purified using the GRS PCR & Gel Band Purification Kit (GRiSP^®^, Porto, Portugal). Bidirectional sequencing using the dideoxy-chain termination method was performed with the appropriate internal primers. Sequence trimming and ClustalW multiple alignment were performed using BioEdit v7.2.5 [56]. The obtained nucleotide consensus sequences were compared with the sequences available in the NCBI (GenBank) nucleotide database (http://blast.ncbi.nlm.nih.gov/Blast, (31 July 2022). Sequence analysis was performed with the phylogenetic tool implemented in MEGA 11 [57], using the maximum-likelihood (ML) method for computing the best fitting substitution model. The Tamura 3-parameter model was selected for all the computed phylogenetic trees. All models were implemented under the assumption that the rate variation among the sites was gamma distributed. Bootstrap consensus trees were inferred from 1000 pseudoreplicates. Phylogenetic trees were edited for style using the Interactive Tree of Life (iTOL) platform [58]. All sequences generated in this study have been deposited in the GenBank database under the accession numbers ON960179-ON960181 (*C. muris; SSU* rRNA), OP964741-OP964760 (*Giardia* spp.; *SSU* rRNA), OP963933-OP963936 (*Giardia* spp.; *bg*) and OP977984-OP977988 (*Giardia* spp.; *gdh*). The accession numbers of each obtained sequence are listed in Table S2.

### 2.7. Mapping and Statistical Analysis

The sampling units, proportion of host species per unit and origin of *Giardia* spp. and *Cryptosporidium* spp.-positive samples were mapped in QGIS (version 3.26.3, Grüt, Swizerland) [59].

To estimate which factors affected the individual probability of *Giardia* spp. infection, a generalized linear mixed effect model (GLMM; using the function “glmer” from the lme4-package) with binomial error distribution was generated for all the small mammal species, where the individual infection status (infected/not infected) was used as a dependent variable. “Species” and “habitat” were set as fixed factors and the “sampling units” were incorporated as a random factor. In cases of samples derived from mixed habitats (e.g., shrubs/olive groves), only the most abundant habitat category (e.g., shrubs) was included. Backward model selection was performed using the “drop1” function. As the results indicated southwestern water voles and Lusitanian pine voles as the main reservoir species, two additional GLMMs were generated to investigate the species-specific habitat effects, where individual infection status was used as a dependent variable and the respective “habitat” was set as a fixed factor and the “sampling unit” was incorporated as a random factor. Model fit was assessed visually using binned residual plots from the arm package.

For all binomial GLMMs, both categorical factors “species” and “habitat” had more than two levels and were, therefore, employed in additional post hoc analyses to compare within-subject contrasts for all levels, using the “glht” function of the multcomp package. All statistical analyses were performed using R (version 4.1.3, Vienna, Austria) [60].

## 3. Results

### 3.1. Occurrence of Giardia spp. and Cryptosporidium spp.

**Figure 1:**
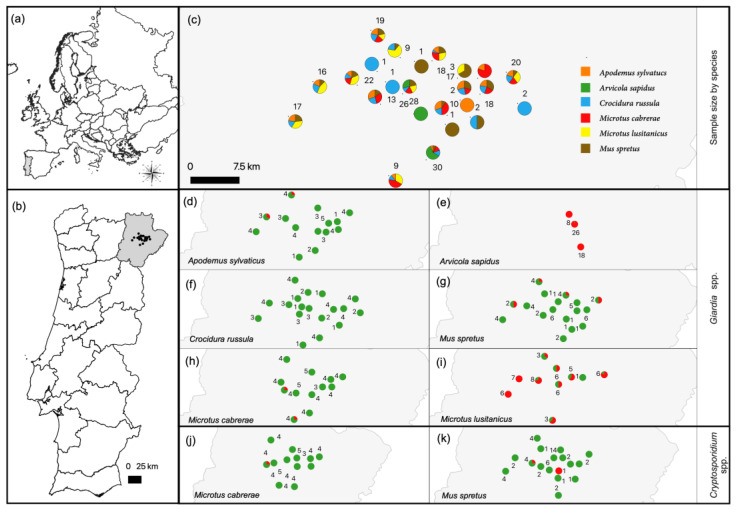
(**a**) Map of Europe. (**b**) Map of Portugal, including the study area in the Trás-os-Montes region, Northeast Portugal. (**c**) Location of the 24 sampling units centered in olive grove patches, proportion of surveyed small mammal species per unit and number of animals per unit. (**d**–**i**) Proportion of *Giardia* spp.-positive (red) and -negative (green) animals per sampling unit for each host species. (**j**,**k**) Proportion of *Cryptosporidium* spp.-positive (red) and -negative (green) animals per sampling unit for *Microtus cabrerae* and *Mus spretus.* Common names of small mammal species are as follows: wood mouse (*Apodemus sylvaticus*), southwestern water vole (*Arvicola sapidus*), greater white-toothed shrew (*Crocidura russula*), Algerian mouse (*Mus spretus*), Cabrera’s vole *(Microtus cabrerae*) and Lusitanian pine vole (*Microtus lusitanicus*).

A total of 95 out of the 290 samples tested were *Giardia* spp.-positive in the *SSU* rRNA gene qPCR assay, representing a general occurrence of 32.8% (95% confidence interval (CI): 27.4–38.5) (Table 1). Analyzing by species, there was a significant difference between the occurrence values of the small mammal species (*p* < 0.001), with values ranging from 0 to 98.1%, being the highest in southwestern water voles (51/52; 98.1%) and Lusitanian pine voles (36/51; 70.6%) and lowest in Algerian mice (4/48; 8.3%), wood mice (2/43; 4.7%) and Cabrera’s voles (2/49; 4.1%). No *Giardia* spp. DNA was detected in greater white-toothed shrews (Table 1). Geographical analysis revealed that the *Giardia* spp.-positive fecal samples were detected in 12 out of 24 sampling units and were distributed over the whole study area (Figure 1).

A total of three out of the 290 samples tested were *Cryptosporidium* spp.-positive in the *SSU* rRNA gene PCR assay, representing a general occurrence of 1% (95% CI: 0.3–9.3). Two positive samples derived from Algerian mice (2/48; 4.2%) and one sample from Cabrera’s vole (1/49; 2%) (Table 1). Regarding the geographical distribution, the three positive samples derived from two non-neighboring sampling units. In one sampling unit, positive samples originated from Cabrera’s vole and Algerian mouse and in the other sampling unit, the sample originated from the remaining Algerian mouse (Figure 1).

### 3.2. Characterization of Giardia spp.

Positive sampling units differed between the host species, due to their unequal distribution over the sampling units (Figure 1). In the full GLMM that included all the host species, the habitat did not explain any variability in infection probability and was excluded as a factor. Thus, the “species” was the only significant factor that affected the probability of *Giardia* spp. infection (Table S3; intercept). Southwestern water voles and Lusitanian pine voles showed the highest infection probabilities (Figure S1; Table S3; postHoc factor “*Species*”). The species-specific analyses testing of only southwestern water voles or Lusitanian pine voles also showed no significant influence of habitat on the infection probability of *Giardia* spp. (Figures S2 and S3; Table S3).

**Figure 2:**
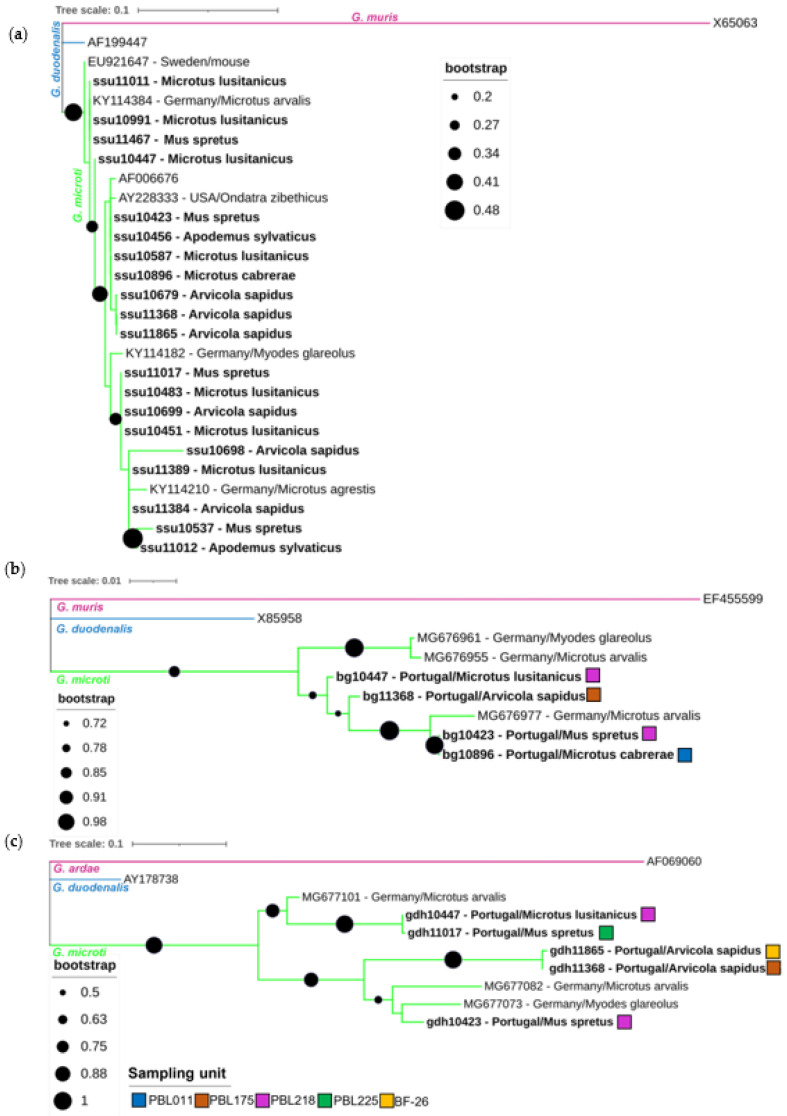
Phylogenetic analysis of *SSU* rRNA (**a**), *bg* (**b**) and *gdh* (**c**) gene sequence fragments of *Giardia microti* characterized in this study (represented in bold) and sequences downloaded from GenBank. Inference was performed using a maximum-likelihood method and the Tamura-3-para-meter model, with discrete gamma distribution (alignment length of *SSU* rRNA: 214 nucleotides; *bg* 478 nucleotides; *gdh* 370 nucleotides). Bootstrap values from 1000 replicates are shown at the nodes and for (**b**,**c**), only bootstrap values above 50% are shown. Analyses were conducted via MEGA X, with editing via the Interactive Tree of Life (iTOL). The reference sequences (GenBank accession numbers) for the *SSU* rRNA gene included *G. muris* (X65063; pink clade), *G. duodenalis* (AF199447, assemblage BIII; blue clade) and *G. microti* (AF006676; green clade). References for *bg* included *G. muris* (EF355599; pink clade) and *G. duodenalis* (X85958, assemblage AI; blue clade) sequences. References for *gdh* included *G. ardae* (AF069060; pink clade) and *G. duodenalis* (AY178738, assemblage B; blue clade) sequences. The *G. microti SSU* rRNA, *bg* and *gdh* sequence fragments obtained from other studies were identified using their accession numbers, country of origin and host. The original sampling units of the corresponding samples are shown as colored squares for (**b**,**c**).

Specific sequences of the *Giardia SSU* rRNA, *bg* and *gdh* genes were determined and compared to reference sequences, if available (Figure 2). Additional sequences from different molecular studies on *Giardia* in rodents were included in all the phylogenetic trees [17,61,62]. Twenty of the selected twenty-four qPCR positive samples were successfully sequenced for the *SSU* rRNA gene fragment and could be assigned to *Giardia* spp. Phylogenetic analysis revealed that all the obtained sequences clustered within the *G. microti* clade, indicating that all the tested rodents were infected with this *Giardia* species (Figure 2a). Within the twenty *G. microti SSU* rRNA gene sequences, five unique sequence variants were identified, which differed in 0–9 single nucleotide polymorphisms (SNPs) from each other over an alignment length of 214 bp, indicating a divergence within the *G. microti* sequences. Comparing the five obtained unique *SSU* rRNA sequences with the published sequences in the GenBank nucleotide database, one sequence was revealed to be novel (ssu10698). Analyses of the five obtained *gdh* sequences and four obtained *bg* sequences support the high genetic divergence of *G. microti* at these loci and existence of supposedly phylogenetically distinct subgroups (Figure 2b,c). Within the four obtained *bg* sequences, three unique sequence types were identified, whereas the five obtained *gdh* sequences were all unique. Comparing the obtained *gdh* and *bg* sequences with the published sequences in the GenBank nucleotide database, all sequences were revealed to be novel. Looking at the sampling units, no spatial clustering of the closely related sequences was observed (e.g., gdh10447 derived from sampling unit PBL218, whereas gdh11017 derived from sampling unit PBL225; Figure 2c). Associations between the phylogenetic subgroups and host distribution could not be examined, due to the low number of *gdh*- and *bg*-positive samples.

### 3.3. Characterization of Cryptosporidium spp.

**Figure 3:**
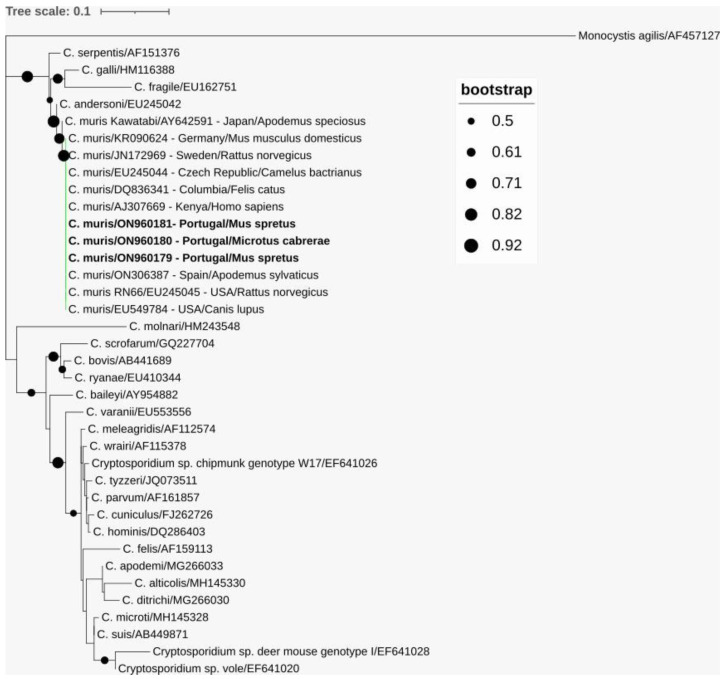
Phylogenetic analysis of *SSU* rRNA gene sequence fragments of *Cryptosporidium muris* characterized in this study (represented in bold) and sequences downloaded from GenBank. Inference was performed using a maximum-likelihood method and the Tamura-3-parameter model with discrete gamma distribution (alignment length: 475 nucleotides). Bootstrap values above 50% from 1000 replicates are shown at the nodes. Analyses were conducted via MEGA X, with editing via the Interactive Tree of Life (iTOL). Sequences are identified with GenBank accession numbers and, additionally, *C. muris* sequences with their country of origin and host. *Monocystis agilis* was used as the outgroup.

The sequence analyses revealed that the three obtained amplicons shared 100% sequence identity with the reference strain *C. muris* RN66 (EU245045) and various other *C. muris* sequences from mammals of different orders worldwide and differed from the *C. muris* Kawatabi strain (AY642591) (Figure 3).

## 4. Discussion

The potential role of wild small mammals as a reservoir of these pathogenic parasites, namely *Giardia* and *Cryptosporidium* spp., remains unknown in Portugal. To fill this knowledge gap, this study provides the first molecular-based evidence on the occurrence, and genetic diversity of *Giardia* and *Cryptosporidium* in wild rodents and shrews in Portugal.

After examining 290 fecal samples from six wild small mammal species from Northeast Portugal, *Giardia* revealed a high occurrence and was detected in 32.8% of the samples. The occurrence values strongly varied between the six examined small mammal species. In European countries, systematic studies on small mammals and rodents in wildlife demonstrate *Giardia* spp. occurrence rates of 2.8% in Spain (in 11 species of small mammals), 7.1–52% in Germany (*Apodemus* spp., *Microtus* spp. and bank voles *Myodes glareolus*) and 24.4–41.7% in Poland (*Apodemus* spp., bank voles) [5,17,19,40]. Hence, in line with this study, rodents are frequently parasitized by *Giardia* spp. In particular, the occurrence values of *Giardia* spp. were higher in southwestern water voles and Lusianian pine voles, when compared to those of Algerian mice, wood mice, Cabrera’s voles and greater white-toothed shrews. Regarding insectivores, molecular studies on *Giardia* spp. are rare. One study from the Netherlands identified *Giardia* spp. in the hedgehog *Erinaceus europaeus* (11% occurrence) [7], whereas other studies also lack the detection of *Giardia* spp. in shrews [5,40]. Nonetheless, care should be taken when comparing occurrence data from different studies, as data were derived from different detection methods, small mammal species, habitats, regions, seasonality and climate conditions.

Phylogenetic analysis of the 20 obtained *SSU* rRNA gene sequence fragments revealed that all the variants clustered within the *G. microti* clade. The low bootstraps values in the *SSU* phylogenetic tree still infer a slight uncertainty in species assignment, but the phylogenetic trees of the *bg* and *gdh* loci of six distinct amplicons show high bootstrap values, and thus strongly support their assignment to *G. microti*. Combining the occurrence values of the conducted qPCR with the phylogenetic results, it can be deduced that southwestern water voles and Lusitanian pine voles are commonly infected with *G. microti* in Northeast Portugal. *G. microti,* as a predominant circulating species in rodents of the family Cricetidae, is known from an early study on wild rodents [16] and was confirmed in several recent studies. *G. microti* was detected in Günther’s vole (*Microtus guentheri*) in Italy [27] and is commonly found in common voles (*Microtus arvalis*), field voles (*Microtus agrestis*) and bank voles in Germany [17]. A study from Sweden reported *G. microti* in yellow-necked field mice (*Apodemus flavicollis*) [63] and another study from Poland reported *G. microti* in common voles and bank voles [64]. To date, *G. microti* is considered as a rodent-adapted species [65] and not thought to infect humans [66]. Thus, the dominant occurrence of *G. microti* and lack of the zoonotic species *G. duodenalis* in this study implies a low potential risk for the transmission of zoonotic *Giardia* species from wild rodents and shrews in Northeast Portugal.

Interestingly, one study suggests the subdivision of *G. microti* into several phylogenetically distinct subgroups, with each subgroup preferentially harbored by a species of a particular rodent host genus [17]. In the present study, the support of nodes of the phylogenetic *SSU* rRNA gene fragment tree and the number of sequenced samples were too low to allow unequivocal identification of possible genetic substructures. However, the high genetic diversity of the sequenced *bg* and *gdh* genes support the potential subdivision of the *G. microti* clade. If the *G. microti* subgroups only circulate within the rodent population of the sampling unit or are spread between different sampling units, they cannot be deduced using a molecular approach, as the obtained number of *gdh* and *bg* sequences would be too low for reliable analysis. Because up to now the deposited *bg* and *gdh* sequences in public databases for *G. microti* were only obtained by one study [17], the present work further enlarges the database and facilitates future comparative studies.

Concerning the methodology for the identification of the *Giardia* species, it should be noted that the typing efficiency at the *bg* and *gdh* loci was lower in comparison to the *SSU* rRNA gene locus. This pattern is in accordance with previous studies [17,40,67] and can be explained by the fact that multicopy genes, such as the *SSU* rRNA gene, show higher sensitivity than single-copy markers (e.g., *bg* and *gdh* genes) [68]. However, single-copy markers should be still employed for genotyping, due to the relatively low discriminatory power of the *SSU* rRNA gene region for *Giardia* spp. [69].

It is striking that, in contrast to other studies, the occurrence values of the examined rodents of the family Muridae (Algerian mice and wood mice) were very low and no other *Giardia* species other than *G. microti* were detected; however, the different host species and habitats of these studies could have influenced the presence of zoonotic *Giardia* [17,25,28,29,63]. Previous molecular studies revealed that small rodents can be infected by *G. microti*, *G. muris* and/or *G. duodenalis* assemblages A/B/G, with *G. microti* associated mainly with rodents of the family Cricetidae (such as voles and muskrats) and *G. muris* linked to the rodent family Muridae [17,25,28,29,63]. Regarding the zoonotic diarrhea-causing protist *G. duodenalis*, the potential risk for transmission from wild rodents to humans is still under debate. Some studies based on accurate genotyping reported *G. duodenalis* as the most frequent species in rodents [21,28,29,70], whereas other studies reported a low proportion of *G. duodenalis*-positive samples and, in line with this study, pointed out the low potential risk for zoonotic transmission [17,26,63]. It is suspected that the greatest zoonotic risk is associated with *Giardia* assemblages that circulate in livestock, but not in free-living wild mammals or their habitats [5,71,72]. Regarding *G. muris*, this species was described extensively in rodents of the family Muridae (yellow-necked field mouse; striped field mouse, *Apodemus agrarius*; wood mouse; and house mouse, *Mus musculus*) [15,17,63], indicating host preferences [15,17]. In this study, rodents of the family Muridae were included (wood mice and Algerian mice), but no *G. muris* was detected in these species. Until now, no other records exist of the detection of *G. muris* in Algerian mice. The applied qPCR assay of this study [51] shows a low sensitivity of *G. muris* detection [25]. Thus, our lack of positive results for *G. muris* and *G. duodenalis* may be related to the applied qPCR assay or relatively low number of sequenced amplicons or to the reduced circulation of these parasites within the rodent community of Northeast Portugal agro-ecosystems.

Overall, the “species” of small mammals was the only significant factor that affected the probability of *Giardia* spp. infection, with the highest occurrence reported in southwestern water voles (98.1%) and Lusitanian pine voles (70.6%). No statistically significant differences were found between habitats and the occurrence of *Giardia* spp. In previous studies, the *Giardia* spp. occurrence also varied between the examined rodent species [5,17,19,28,40,73]. Interestingly, a study from Germany associated lower occurrence values in *Apodemus* spp. with a lower abundance of *Giardia* cysts in individuals of this rodent genus, compared to *Microtus* spp. and bank voles [17]. In this study, the *Giardia* spp. abundance was derived from cyst numbers in fecal samples and parasite DNA abundance in feces, estimated by the Ct values of the qPCR results. In the present study, not enough Ct values of the rodent species with low *Giardia* spp. occurrence (wood mice, Cabrera’s voles and Algerian mice) are available to make meaningful comparisons with the Ct values of the species with high *Giardia* spp. occurrence (southwestern water voles and Lusitanian pine voles).

Concerning the other factors (habitats and sampling units), analyses on this topic from other studies are rare, resulting in the poor identification of the additional drivers of *Giardia* spp. occurrence in rodents. One study from Poland found, in accordance with the present study, no significant difference when comparing the study sites/vegetation and *Giardia* spp. occurrence in striped field mouse, yellow-necked field mouse and bank vole [19]. Another study from Poland pointed out that extrinsic factors (such as annual and seasonal fluctuations in rodent po-pulations) play a more important role than intrinsic factors (such as age and sex in the rodent individuals) in the ecology and occurrence of *Giardia* and this is also the case for the occurrence of *Cryptosporidium* and other enteric protozoa [74]. It was observed that *Giardia* spp. infection peaked more frequently in spring or in autumn, which is when, in contrast to summertime, the temperature and humidity conditions are more suitable for the survival of protozoan cysts in the environment, creating conditions for the enhanced efficiency of transmission [19,74]. In the present study, sampling took place in May–July 2022, possibly favoring a higher occurrence of *Giardia* spp. Nonetheless, the statistical results in this study should be interpreted carefully, as not all of the host species were represented in all the habitat categories. Hence, although no statistically significant differences between habitats and the occurrence of *Giardia* spp. were found, the remarkably high infection rate of southwestern water voles may be related to its habitat preferences. This species is strongly linked to water streams and ponds with a high cover of grassy vegetation [75,76]. In water, *Giardia* spp. can persist and remain infective as robust cysts [43] and the waterborne route (contaminated drinking water) is probably the most widely recognized means of *Giardia* transmission to cause human giardiasis [66]. The sources of drinking water include rivers, reservoirs, canals, or lowland reservoirs and the *Giardia* cysts probably enter these surface waters from agricultural or urban runoff, wastewater treatment discharges or biosolids [43]. As southwestern water voles live close to untreated surface water, their habitat preference may favor the probability of infection. On the other hand, while Cabrera’s voles are also associated with wet habitats (perennial mixed grasses) [48], the *Giardia* spp. infection rate was low for this species. Further environmental and host factors, such as the susceptibility for *Giardia* infection and behavioral patterns (e.g., diet and water consumption), might influence the probability of infection. We acknowledge that more detailed data about the intrinsic host factors (e.g., age and sex) and extrinsic factors (e.g., population size and seasonality effects) are needed to better understand the processes that drive the ecology of *Giardia* transmission within small mammal populations.

*Cryptosporidium* spp. was found only sporadically, with an overall frequency of 1%. Two positive samples from Algerian mice were reported (2/48; 4.2%) and one sample from Cabrera’s vole (1/49; 2%). Hence, *Cryptosporidium* spp. might occur only sporadically in wild small mammals of Northeast Portugal and/or the examined rodent species may represent minor hosts for this eukaryotic parasite. Another possibility for these results could be related to the seasonal differences in fecal shedding. In European studies, *Cryptosporidium* spp. has been detected in nineteen small mammal species from nine different genera from agricultural and forestry environments [40] and the averaged infection rate was 28% [41]. However, recorded occurrence values are highly variable within and between host genera. In *Apodemus* spp., *Cryptosporidium* occurrence ranged from 21% to 68% [19,77], in *Microtus* spp. and *Myodes* spp., it ranged from 2% to 73% [2,19,74,78,79,80,81], in *Mus* spp., it ranged from 0% to 32%, in *Rattus* spp., it ranged from 14% to 45% and in shrews of the genus *Sorex,* it ranged from 14% to 44% [2,8,38,82,83,84]. Thus, in this study, the overall occurrence of 1% and host-specific occurrence values are comparatively lower than in previous studies from Europe. Interestingly, a recent study from Spain that surveyed small mammals in a geographically close region (200 km distance) reported comparable low infection rates of 3.7% overall, suggesting the low occurrence of *Cryptosporidium* spp. in small mammals in the northwest region of the Iberian Peninsula. Nonetheless, occurrence values should be compared carefully as they are affected by many factors, such as the methods used for detection [19,41,85]. Additional studies that include diverse rodent species and regions are required to properly assess the occurrence of *Cryptosporidium* spp. in wild small mammals from Portugal.

*Cryptosporidium muris* was identified as the only *Cryptosporidium* species in two samples of Algerian mice and one sample of Cabrera’s vole from geographically separated sampling units. As expected, the obtained *SSU* rRNA gene sequences shared 100% sequence identity with the reference strain *C. muris* RN66, the most frequently detected strain in Europe [63,77,86,87,88]. In future studies, additional genetic markers, such as microsatellite markers, could be added to provide a more detailed understanding of the genetic variability in *C. muris* [89]. *C. muris* has been described in a variety of mammals and up to now, it has been identified in 17 rodent species worldwide [41]. In the European scenario, *C. muris* was identified in yellow-necked field mice, wood mice, house mice, Algerian mice, bank voles, black rats (*Rattus rattus*) and brown rats (*Rattus norvegicus*) [8,40,77,82,83] and Cabrera’s voles in the present study. Moreover, *C. muris* is considered as a zoonotic species [85], since it has been reported in healthy children [90] and HIV-positive individuals [91,92,93,94] and also healthy adults were susceptible to experimental infection [95]. As rodents have free access to water resources and frequent contact with various domestic animals (i.e., sheep, cattle and goats) [42], the detection of *C. muris* in rodents may present a potential risk for public and animal health. Nonetheless, the low occurrence in this study implies the overall limited role of rodents as a natural source for *Cryptosporidium* in Northeast Portugal.

We did not detect any types of *Cryptosporidium* spp. other than *C. muris* in the examined host species. As the number of positive samples was very low, no reliable statement about the diversity of *Cryptosporidium* species/genotypes that circulate in wild small mammals in Portugal can be given. Previous European studies recorded a total of 15 species and 16 genotypes of *Cryptosporidium* [40,41,42]. In the neighboring country Spain, most of the *Cryptosporidium* strains in surveyed small mammals corresponded to rodent-adapted species (*C. ditrichi, C. muris and C. tyzzeri*) or genotypes (rat genotypes CR97 and W19; vole genotypes V and VII); however, *C. parvum* was also detected [40]. The same pattern was observed for insectivores, which are mainly infected with adapted species and genotypes (*C. erinacei* and shrew genotypes I and II) [2,96]. The frequency of *C. parvum*, a species with loose host specificity and zoonotic risk, has been evaluated to be different for rodents in European countries. A review considers *C. parvum* as the dominant species in rodents in Europe [41], whereas others suggest that natural *C. parvum* infections are relatively rare in rodents [40]. However, both studies suggest that free-living small mammals in agricultural areas are potential reservoirs of *C. parvum,* and thus may play an important role in the ecology of the zoonosis [40,41]. The lack of *C. parvum* in this study may be partially explained by the fact that the collected samples originate from a landscape that is largely dominated by traditional olive groves [48] with few livestock farms, where *C. parvum* is responsible for most of the cattle infections [97]. Future studies that compare rodents from wildlife and cattle farms could be useful to examine the occurrence of *C. parvum* and other *Cryptosporidium* species in these different target regions in Portugal and gain an understanding of the possible transmission cycles.

The interpretation of the results of our study has some limitations that should be overcome in future studies. As an initial approach, we diluted the DNA by pooling the samples, resulting in lower concentrations, perhaps below the limit of detection. This might be a problem in the case of *Cryptosporidium* detection, as in this study, a conventional PCR approach was followed. The results of the PCR investigations might be also hampered by the lack of a (internal) extraction and amplification control, preventing the evaluation of the efficiency of DNA extraction and PCR amplification. The limitation of the MLST to circa 15% of the *Giardia* spp.-positive samples and their selection based on the Ct value of the qPCR might also result in bias for the detection of *G. microti*, although other *Giardia* species could be circulating in these animals. Moreover, given that a sample contains several parasite species of different loads, dideoxy-chain termination sequencing and subsequent consensus sequence generation can cause biased results and a failure in the detection of minor parasite species or subtypes. Future studies should employ next-generation sequencing approaches to overcome the limitations mentioned here and to allow a better characterization of the diversity of these parasites.

## 5. Conclusions

This study reports the occurrence of *Giardia* spp. and *Cryptosporidium* spp. in wild rodents and shrews in Portugal. The diversity of *Giardia* and *Cryptosporidium* species was low, as only *C. muris* and *G. microti* were identified. *C. muris* was found sporadically, whereas rodent-adapted *G. microti* was frequently detected in cricetid hosts. Therefore, our findings suggest the limited role of small mammals as natural sources of human infections in Northeast Portugal regarding the investigated parasites. Moreover, molecular evidence is provided for the high genetic diversity within the *G. microti* clade and the database of sequences relevant for the sequence typing of *G. microti* strains is further expanded. In addition, this is the first record of *G. microti* in southwestern water voles, Lusitanian pine voles, Algerian mice, wood mice and Cabrera’s voles, and *C. muris* in Cabrera’s voles. Both *Giardia* and *Cryptosporidium* spp. were not detected in greater white-toothed shrews. Additional investigations are required to elucidate the epidemiology of *Giardia* spp. and *Cryptosporidium* spp. in wild small mammals in Portugal and their possible public health repercussions.

## Figures and Tables

**Table 1:** Occurrence of *Cryptosporidium* spp. and *Giardia* spp. in small mammals analyzed using the *SSU* rRNA gene PCR and qPCR assays, respectively, and the results of *Giardia microti* genotyping at the *SSU* rRNA, *bg* and *gdh* gene loci.

**Animals**		**Results of Screening** No. of positive samples/No. of analyzed samples (Occurrence in % and 95% CI)		**Results of Genotyping of Selected *Giardia* spp.-Positive Samples** No. of positive samples/No. of analyzed samples		
Family	Species (common name)	*Cryptosporidium* spp. (*SSU* rRNA PCR)	*Giardia* spp. (*SSU* rRNA qPCR)	*G. microti* (*SSU* rRNA PCR)	*G. microti* (*bg* PCR)	*G. microti* (*gdh* PCR)
Muridae	*Apodemus sylvaticus* (wood mouse)	0/43	2/43 (4.65%; 0.01–15.81)	2/2	0/2	0/2
	*Mus spretus* (Algerian mouse)	2/48 (4.17%; 0.00–9.82)	4/48 (8.33%; 2.32–19.98)	4/4	1/4	2/4
Total		2/91 (2.20%; 0.27–7.71)	6/91 (6.60%; 2.46–13.80)	6/6	1/6	2/6
Cricetidae	*Arvicola sapidus* (southwestern water vole)	0/52	51/52 (98.08%; 89.74–99.95)	6/8	1/8	2/8
	*Microtus cabrerae* (Cabrera’s vole)	1/49 (2.04%; 0.00–10.85)	2/49 (4.08%; 0.01–13.98)	1/2	1/2	0/2
	*Microtus lusitanicus* (Lusitanian pine vole)	0/51	36/51 (70.59%; 56.17–82.51)	7/8	1/8	1/8
Total		1/152 (0.66%; 0.00–3.61)	89/152 (58.55%; 50.29–66.48)	14/18	3/18	3/18
Soricidae	*Crocidura russula* (greater white-toothed shrew)	0/47	0/47	-	-	-
Total		3/290 (1.03%; 0.21–2.99)	95/290 (32.76%; 27.39–38.49)	20/24	4/24	5/24

*bg*—beta-giardin; C—*Cryptosporidium*; CI—confidence interval; *G*—*Giardia*; *gdh*—glutamate dehydrogenase; *No.*—number; *SSU*—small subunit.

## Data Availability

All the results of the study are presented within the manuscript and its Supplementary files.

## Supplementary Materials

The following supporting information can be downloaded at: https://www.mdpi.com/article/10.3390/ani13030515/s1, Table S1: Oligonucleotides used for the molecular identification and/or characterization of *Cryptosporidium* spp. and *Giardia* spp. in the present study; Table S2: Accession numbers of nucleic acid sequences generated in the present study, their phylogenetic assignment, and their highest identity sequence in BLAST; Table S3: Results of generalized linear mixed model (intercept) and post hoc analyses (postHoc factor), showing the influence of small mammal species on the *Giardia* spp. infection probability (Sheet 1) and the influence of habitats on the *Giardia* spp. infection probability of southwestern water voles (*Arvicola sapidus*) (Sheet 2) and Lusitanian pine voles (*Microtus lusitanicus*) (Sheet 3). The sampling units were incorporated as random factors in all the models and the factor habitat was excluded from the Sheet1 models based on model selection; Figure S1: Mean results (+/- 95% confidence interval) of binomial generalized linear mixed model that shows the influence of small mammal species on the *Giardia* spp. infection probability. The sampling units were incorporated as random factors and habitats were excluded from the model during the selection process; Figure S2: Results of generalized linear mixed models that show the influence of habitats on the *Giardia* spp. infection probability of southwestern water voles (*Arvicola sapidus*). The sampling units were incorporated as random factors; Figure S3: Results of generalized linear mixed models that show the influence of habitats on the *Giardia* spp. infection probability in Lusitanian pine voles (*Microtus lusitanicus*). The sampling units were incorporated as random factors.
