# Supplementary material for: Detection and Molecular Characterization of Giardia and Cryptosporidium spp. Circulating in Wild Small Mammals from Portugal
Source: Animals (Basel). 2023 Feb 1;13(3):515. doi: 10.3390/ani13030515 (PMC9913638; doi:10.3390/ani13030515)
Supplement: Supplementary file 1 [file animals-13-00515-s001.zip › Table S1_20221205.pdf]

animals

MDPI

Table S1. Oligonucleotides used for the molecular identification and/or characterization of *Cryptosporidium* spp. and *Giardia* spp. in the present study.

Target organism	Locus	Oligonucleotides	Sequence (5′→ 3′)	Reference
Cryptosporidium spp.	*SSU* rRNA	CR-P1	CAGGGAGGTAGTGACAAGAA	[55]
		CR-P2	TCAGCCTTGCGACCATACTC	
		CR-P3	ATTGGAGGGCAAGTCTGGTG	
		CPB-DIAGR	TAAGGTGCTGAAGGAGTAAGG	
Giardia spp.	*SSU* rRNA	Probe	FAM–CCCGCGGCGGTCCCTGCTAG–BHQ1	[51]
		Gd-80F	GACGGCTCAGGACAACGGTT	
		Gd-127R	TTGCCAGCGGTGTCCG	
	SSU rRNA	RH11-derivates	Equal mix of CATCCGGTCGATCCTGCC and CATCCGGTTGATCCTGCC	[17]
		Gia2150c	CTGCTGCCGTCCTTGGATGT	
		RH-4 derivates	Equal mix of AGTCGAACCCTGATTCTCCGCCAGG and AGTCAAACCCTGATCCTCCGCCAGG and AGTCGAACCCTGATTCTCCGTCAGG	
	gdh	GDHeF	TCAACGTYAAYCGYGGYTTCCGT	[53]
		GDHiR	GTTRTCCTTGCACATCTCC	
		GDHiF	CAGTACAACTCYGCTCTCGG	
	bg	G7	AAGCCCGACGACCT CACCCGCAGTGC	[54]
		G759	AGGCCGCCCTGGATCTTCGAGACGAC	
		G99	GAACGAACGAGATCGAGGTCCG	
		G609	CTCGACGAGCTTCGTGTT	

bg — beta-giardin; *gdh* — glutamate dehydrogenase; *SSU* — small subunit
