# Supplementary figures and images for: Detection and Molecular Characterization of Giardia and Cryptosporidium spp. Circulating in Wild Small Mammals from Portugal
Source: Animals (Basel). 2023 Feb 1;13(3):515. doi: 10.3390/ani13030515 (PMC9913638; doi:10.3390/ani13030515)

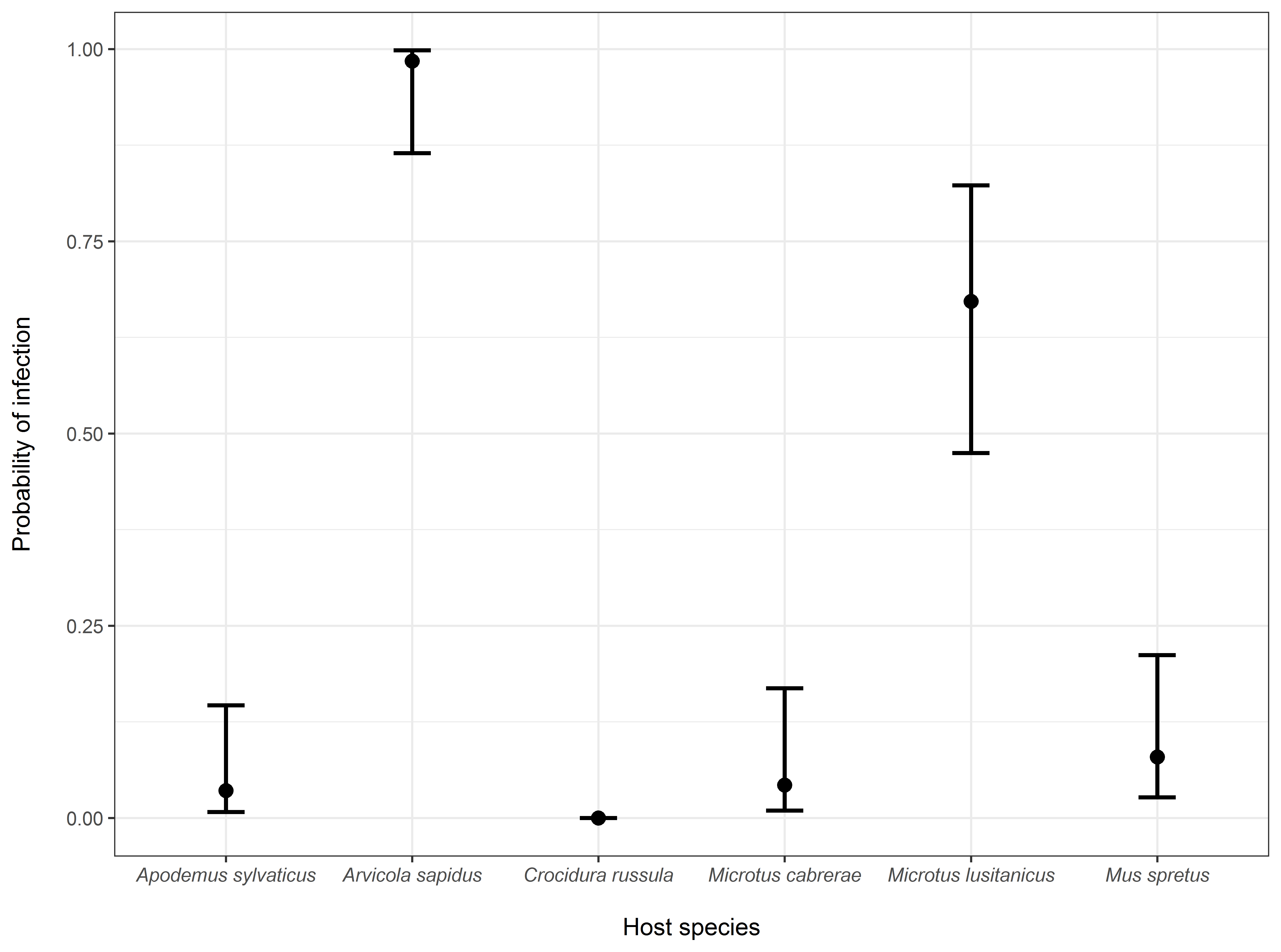

Supplement: Supplementary file 1 [file animals-13-00515-s001.zip › Figure S1.tiff]

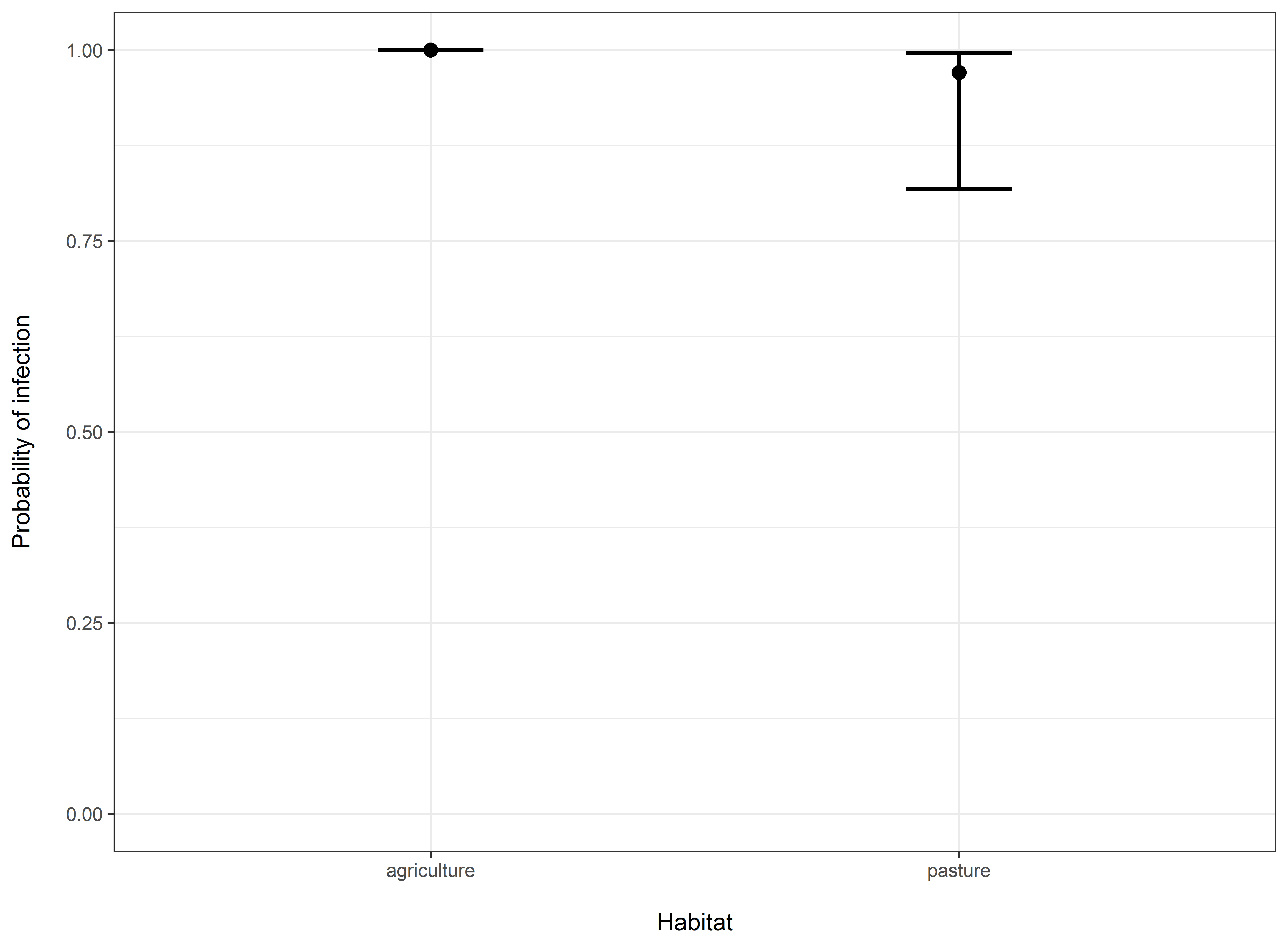

Supplement: Supplementary file 1 [file animals-13-00515-s001.zip › Figure S2.tiff]

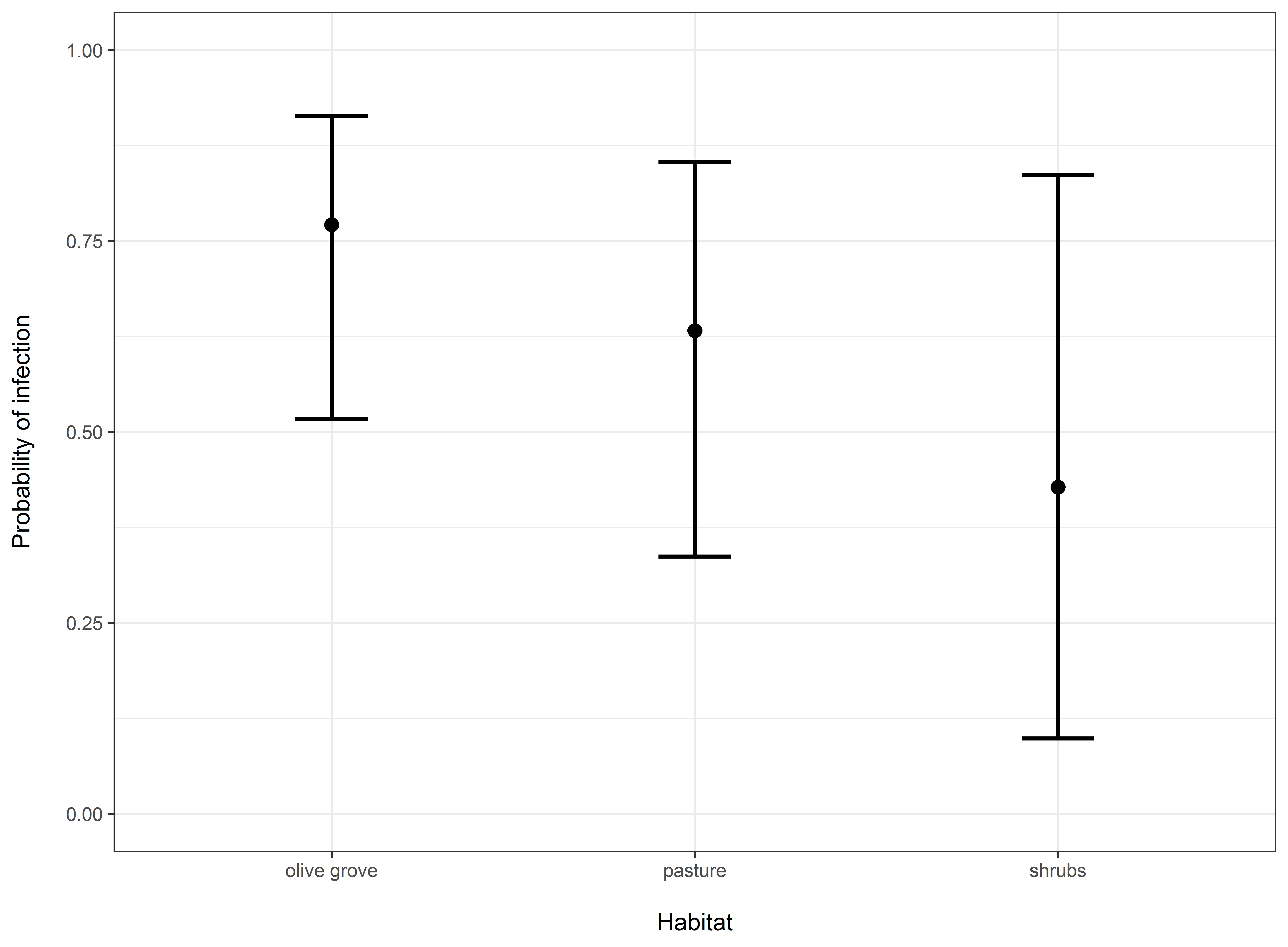

Supplement: Supplementary file 1 [file animals-13-00515-s001.zip › Figure S3.tiff]
